# Clinical study of Dysfunctional Ventilatory Weaning Response in critically ill patients*

**DOI:** 10.1590/1518-8345.3522.3334

**Published:** 2020-08-12

**Authors:** Ludmila Christiane Rosa da Silva, Isadora Soto Tonelli, Raissa Caroline Costa Oliveira, Patricia Lage Lemos, Selme Silqueira de Matos, Tania Couto Machado Chianca

**Affiliations:** 1 Universidade Federal de Minas Gerais, Escola de Enfermagem, Belo Horizonte MG, Brazil.; 2 Scholarship holder at the Coordenação de Aperfeiçoamento de Pessoal de Nível Superior (CAPES), Brazil.; 3 UNIMED, Unidade de Terapia Intensiva, Belo Horizonte, MG, Brazil.; 4 Universidade Federal de Minas Gerais, Hospital Risoleta Tolentino Neves, Belo Horizonte, MG, Brazil.

**Keywords:** Ventilador Weaning, Treatment Failure, Intensive Care Units, Nursing, Nursing Diagnosis, Validation Studies, Desmame do Respirador, Falha de Tratamento, Unidades de Terapia Intensiva, Enfermagem, Diagnóstico de Enfermagem, Estudos de Validação, Desconexión del Ventilador, Insuficiencia del Tratamiento, Unidades de Cuidados Intensivos, Enfermería, Diagnóstico de Enfermería, Estudios de Validación

## Abstract

**Objective:**

to clinically validate the nursing diagnosis of *Dysfunctional Ventilatory Weaning Response* in adult patients admitted to Intensive Care Units.

**Method:**

a concurrent cohort performed with 93 patients admitted to Intensive Care Units. The incidence and incidence density of the diagnosis were estimated, its related factors were identified based on bivariate analysis and clinical indicators for determining its occurrence, according to the global and temporal presentation.

**Results:**

the overall incidence of the diagnosis was 44.09% and the incidence density was 14.49 occurrences for every 100 extubations/day. The factors related to the diagnosis were the following: age, clinical severity, fluid balance, oliguria, hemodialysis, edema in upper/lower limbs, anasarca, number of antibiotics, hypothermia, hyperthermia, amount of secretion, muscle retraction, anxiety score, heart rate, use of vasopressors and non-invasive ventilation after extubation. The clinical indicators most frequently identified for determining the diagnosis were the following: tachypnea, drop of saturation and tachycardia. Temporal progression in the severity of these manifestations was found.

**Conclusion:**

the *Dysfunctional Ventilatory Weaning Response* is a common finding in critically ill patients. Some components of the diagnosis of the NANDA-International (2018) version could be clinically validated. It is noteworthy that there are variables not yet described in the taxonomy, demonstrating the need to review this nursing diagnosis.

## Abstract (pt)

**Objetivo:**

validar clinicamente o diagnóstico de enfermagem *Resposta Disfuncional ao Desmame Ventilatório* em pacientes adultos internados em Unidades de Terapia Intensiva.

**Método:**

coorte concorrente realizada com 93 pacientes internados em Unidades de Terapia Intensiva. Foram estimadas a incidência e a densidade de incidência do diagnóstico, identificados seus fatores relacionados a partir de análise bivariada e indicadores clínicos de determinação da sua ocorrência, segundo apresentação global e temporal.

**Resultados:**

a incidência global do diagnóstico foi de 44,09% e a densidade de incidência de 14,49 ocorrências a cada 100 extubações/dia. Os fatores relacionados ao diagnóstico foram: idade, gravidade clínica, balanço hídrico, oligúria, hemodiálise, edema em membros superiores/inferiores, anasarca, número de antibióticos, hipotermia, hipertermia, quantidade de secreção, retração muscular, escore de ansiedade, frequência cardíaca, uso de vasopressores e ventilação não invasiva após a extubação. Os indicadores clínicos identificados mais frequentemente para a determinação do diagnóstico foram: taquipneia, queda de saturação e taquicardia. Verificou-se progressão temporal da gravidade dessas manifestações.

**Conclusão:**

a *Resposta Disfuncional ao Desmame Ventilatório* é um achado comum em pacientes críticos. Alguns componentes do diagnóstico da versão NANDA-International (2018) puderam ser validados clinicamente. Destaca-se que existem variáveis ainda não descritas na taxonomia, demonstrando a necessidade de revisão desse diagnóstico de enfermagem.

## Abstract (es)

**Objetivo:**

validar clínicamente el diagnóstico de enfermería *Respuesta Ventilatoria Disfuncional al Destete* en pacientes adultos internados en Unidades de Cuidados Intensivos.

**Método:**

cohorte concurrente realizada con 93 pacientes internados en Unidades de Cuidados Intensivos. Se estimó la incidencia y densidad de incidencia del diagnóstico y se identificaron los factores vinculados a partir de análisis bivariados e indicadores clínicos para determinar su ocurrencia, de acuerdo con la presentación global y temporal.

**Resultados:**

la incidencia global del diagnóstico fue de 44,09% y la densidad de incidencia de 14,49 casos por cada 100 extubaciones/día. Los factores relacionados con el diagnóstico fueron: edad, gravedad clínica, balance hídrico, oliguria, hemodiálisis, edema en miembros superiores/inferiores, anasarca, número de antibióticos, hipotermia, hipertermia, cantidad de secreción, retracción muscular, grado de ansiedad, frecuencia cardíaca, uso de vasopresores y ventilación no invasiva después de la extubación. Los indicadores clínicos identificados con mayor frecuencia para determinar el diagnóstico fueron: taquipnea, disminución de la saturación de oxígeno y taquicardia. Se verificó una progresión temporal en la gravedad de estas afecciones.

**Conclusión:**

La *Respuesta Ventilatoria Disfuncional al Destete* es un hallazgo común en pacientes críticos. Algunos componentes del diagnóstico de la versión NANDA-International (2018) podrían validarse clínicamente. Es de destacar que hay variables aún no descriptas en la taxonomía, lo que demuestra la necesidad de revisar este diagnóstico de enfermería.

## Introduction

Mechanical Ventilation (MV) is a therapeutic support often used in Intensive Care Units (ICU). Multi-center cross-sectional studies demonstrate that up to 46% of the patients admitted to these units require MV at some point during their hospitalization^(^ 1 ^-^ 3 ^)^. However, despite being a primary intervention for the patient with acute or chronic-acute respiratory failure, reducing the work of the respiratory muscles, and reversing or preventing muscle fatigue^(^ 2 ^-^ 3 ^)^, mechanical ventilatory support is capable of inducing several complications, such as Ventilator Associated Pneumonia (VAP)^(^ 4 ^)^, diaphragmatic dysfunction induced by MV^(^ 5 ^-^ 6 ^)^, and critical illness polyneuropathy^(^ 7 ^)^, increasing the morbidity and mortality of a critically ill patient^(^ 8 ^-^ 10 ^)^

In this context, it is essential to reduce the time in which the patient is under invasive artificial ventilation, restoring spontaneous ventilation as soon as possible, a process called ventilatory weaning^(^ 9 ^,^ 11 ^-^ 12 ^)^. The performance of the nurse in this context aims to minimize the adverse events caused by MV and potential risks to which the patient on artificial ventilation is exposed, such as self-extubation, the occurrence of VAP and other nosocomial infections, in addition to extubation failure^(^ 13 ^)^.

The growing increase in the incidence of mechanical ventilatory support in intensive care has driven the interest and development of studies to improve the conduct of this process, in order to minimize the possible negative repercussions related to the prolonged use of MV. In this sense, the occurrence, the monitoring requirements, and the complications related to MV point the responses to ventilatory weaning as relevant in health practice.

Since 1992, NANDA-International (NANDA-I) has established the Nursing Diagnosis (ND) of *Dysfunctional Ventilatory Weaning Response* (DVWR). This ND refers to the activity/rest domain, being defined as “Inability to adjust to decreased levels of mechanical ventilatory support, which interrupts and prolongs the weaning process”^(^ 14 ^)^. However, despite being accepted by NANDA-I about 25 years ago, this ND is not supported by strong scientific evidence and its defining characteristics and related factors are not yet determined in terms of relevance.

Besides, it is considered that the factors related to the diagnosis, in addition to being limited, are outdated in relation to the current scientific production on the human problem/response. It should be noted that validation or review studies of its components have not been identified. Finally, it is worth mentioning that this is one of the ND for which NANDA-I recommends that studies be conducted to produce scientific evidence of the diagnosis itself and its components^(^ 14 ^)^.

Thus, considering the importance of the efficient performance of nurses in the care of critical weaning patients on MV who are candidates for extubation, it is questioned whether the defining characteristics and related factors proposed by NANDA-I for the ND of DVWR are identified in a sample of adult patients admitted to the ICU, undergoing ventilatory weaning.

The present study aims to clinically validate the nursing diagnosis of *Dysfunctional Ventilatory Weaning Response* in adult patients admitted to Intensive Care Units.

## Method

This is a multi-center concurrent cohort study with consecutive follow-up of hospitalized patients in four adult ICUs of two large teaching hospitals in a Brazilian capital. In addition to estimating the incidence and incidence density of DVWR in the sample, the clinical indicators for determining this ND (defining characteristics) and factors related to its occurrence were also observed.

For sample composition, the following inclusion criteria were established: age equal to or greater than 18 years old, remaining on MV by means of an orotracheal tube (OT) for a time equal to or greater than 48 hours, starting weaning from MV on ventilatory support mode and consenting to participate in the research or to have their participation authorized by the responsible person by signing the Free and Informed Consent Form (FICF). Patients were excluded who were diagnosed with neuropathic diseases, such as myasthenia, recent neurological, traumatic, ischemic or hemorrhagic events, as these conditions can impair respiratory drive^(^ 15 ^)^; with occurrence of inadvertent (unplanned) extubation; failure of previous extubation in this hospitalization (reintubation); tracheostomy (TCT); extubations performed in the absence of the research team; death or transfer before the end of the ventilatory weaning process.

Data collection was preceded by a pilot study conducted with 30 patients, to perform the sample calculation, test and refine the data collection instruments. For the sample calculation, the technique of estimating the punctual percentage of patients with failed weaning was performed using the infinite population formula, using conservative criteria^(^ 16 ^)^. The sample size was calculated considering a sampling error (d) of 10%, a confidence (α2z) of 90% in estimating this probability and the incidence of patients who had the DVWR outcome in the pilot study. Thus, considering that, of the 30 patients observed, 16 were found to have the outcome, the incidence of DVWR in the pilot study was 53%, resulting in a calculation of a minimum sample of 68 patients for the original study.

**Figure 1:**
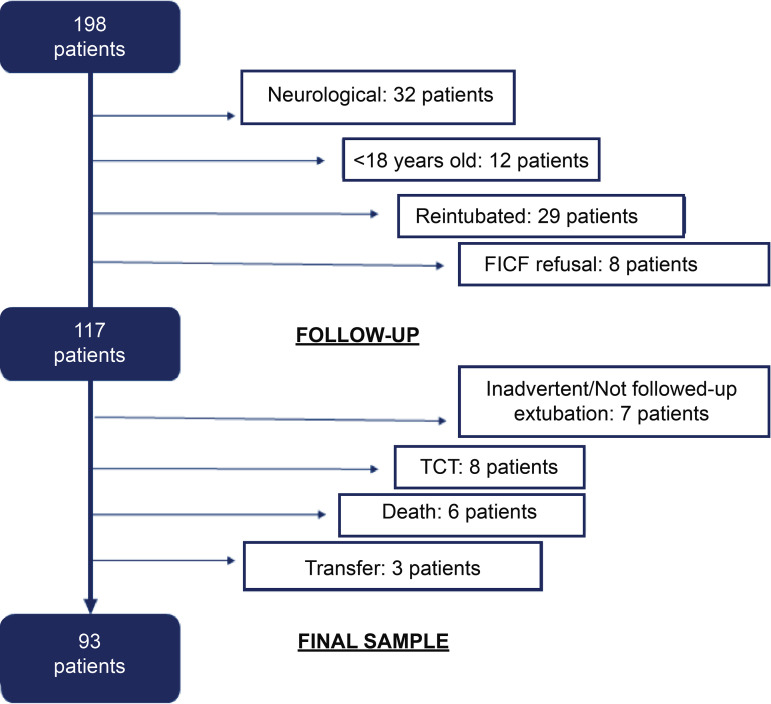
Flowchart of the study sample selection

The clinical research stage, including the pilot study, took place during the period from August 2015 to August 2016. Of the population of 198 patients admitted during the period, 117 were followed up during the clinical stage. After calculating the individuals who fit into situations considered to be lost to follow-up, 93 patients made up the final sample (Figure 1).

Individuals who stayed for more than 48 hours submitted to MV through OT, hospitalized in the study ICU, were tracked daily for the possibility of starting ventilatory weaning and compliance with the other inclusion criteria. Insertion in the study could occur within 24 hours after changing the ventilatory mode, considering the substitution of a controlled mode for a spontaneous one as a milestone to start weaning.

Data collection was carried out every day of the week by the research team, until the patient developed the outcome, was discharged from the ICU, was transferred or died. This collection was carried out through the consecutive monitoring of individuals included in the study and consisted of obtaining daily clinical data concerning the physical examination of the patient, as well as clinical and socio-demographic information available in the medical records, and results of laboratory and imaging tests.

The data collection instruments were built from the components of the ND of DVWR described in the taxonomy, in addition to variables identified in an integrative literature review (ILR) carried out previously. The “INITIAL ASSESSMENT” instrument, applied when the patient was included in the study, contained information such as sociodemographic (gender and age) and clinical (date of admission to the hospital and ICU, origin, primary medical diagnosis, comorbidities, type of hospitalization, the clinical severity indicator - Simplified Acute Physiology Score - SAPS 3 at admission, surgery, time of postoperative period, start of MV, reason for using MV, previous attempt to wean, accumulated fluid balance, occurrence of oliguria, need for hemodialysis, use of antibiotics-type and dose, use of vasopressors-type and dose, use of sedation-type and dose, occurrence of hypothermia or fever in the last 24 hours and laboratory tests) data.

In the days following the initial assessment, the “DAILY FOLLOW UP” instrument was used, which contained variables such as: MV time, accumulated fluid balance, occurrence of oliguria, need for hemodialysis, use of antibiotics-type and dose, use of vasopressors-type and dose, use of sedation-type and dose, occurrence of hypothermia or fever in the last 24 hours, and laboratory tests.

On all the follow-up days, the patients were also evaluated according to the “patient assessment” instrument, which contained information such as: degree of head elevation; level of consciousness according to the Glasgow Coma Scale; sedation level according to the Richmond Agitation-Sedation Scale (RASS); presence and level of edema; occurrence of anasarca; presence of intercostal retraction; quantity and characterization of secretion in OT; the reduced State-Trait Anxiety Inventory-STAI-E; vital signs: heart rate, respiratory rate, mean arterial pressure, peripheral oxygen saturation and axillary, esophageal or tympanic temperature; respirator brand; respiratory parameters: Fraction of Inspired Oxygen (FiO_2_), Positive End-Expiratory Pressure (PEEP), Volume Support Pressure, Tidal Volume (VT), Minute Volume (Vm) and Oxygenation Index (PaO_2_/FiO_2_).

In situations in which the patients were submitted to the Spontaneous Breathing Trial (SBT), characteristics of its performance and its outcome were observed using the “TRIAL” instrument, such as: duration of sedation suspension before the test, duration of the test, previous SBT, way of performing the test, rapid and shallow breathing index, ventilatory parameters used in the SBT, SBT result, signs of SBT failure, time until SBT failure.

Finally, the extubation event marked the last day of the monitoring in the study. On this occasion, during the first three hours after the removal of the artificial airway, the patients were directly observed by the research team regarding the presence of clinical indicators of possible failure of the process, recorded according to their temporal occurrence in the “EXTUBATION” instrument. The following variables were observed: sedation suspension time, extubation outcome, signs of extubation failure and respective temporality, time until extubation failure occurrence, reason for extubation failure, signs of extubation failure, use of Noninvasive Ventilation (NIV) and its purpose.

It is worth mentioning that, for this study, failure to wean was considered as the need for restitution of artificial ventilation and reintubation within 48 hours after extubation, as established in the III Brazilian Consensus on Mechanical Ventilation^(^ 9 ^)^ and in the Brazilian Mechanical Ventilation Guidelines^(^ 12 ^)^.

Data was tabulated in the Epi Info program, version 3.5.1, using double entry. Subsequently, they were exported, processed, and analyzed with the aid of the R software, version 3.3.1.

Descriptive analyses were performed using simple frequency, measures of central tendency (mean and median) and measures of variability (standard deviation and quartiles), presented according to the normal distribution pattern. The overall incidence rates and incidence density of DVWR were determined.

To analyze the association of the possible factors related to the occurrence of the ND, bivariate analysis was used to build the Logistic Regression Model. For the Forward method, a significance level of 25% was adopted. For the Backward method, a 5% significance level was adopted. To verify the association between the occurrence of the diagnosis and the variables that did not enter the regression model, the Chi-Square and Fischer’s Exact tests were used for the qualitative variables and the Mann-Whitney test for the quantitative variables. Thus, the relation between each independent variable and the DVWR outcome variable was obtained, and the risk of DVWR occurrence was measured using Odds Ratio (OR) and its 95% Confidence Interval (CI).

The clinical indicators for determining DVWR were analyzed for the frequency of occurrence in the patients globally and according to their temporal presentation. These factors were observed for three hours after extubation, a period considered critical for the occurrence of failures^(^ 17 ^)^ and recorded every 30 minutes, making 6 observation times (T1-T6).

The study complies with Resolution 466/12, which covers clinical research studies with human beings. The project was sent to the Ethics and Research Committee of the Federal University of Minas Gerais and to co-participating institutions and obtained a favorable opinion under CAAE protocol No. 19684414.0.0000.5149.

## Results

Among the 93 patients extubated in the period, 41 cases of DVWR occurrence were identified. The overall incidence of DVWR was 44.09%. Considering the sum of periods of 283 “People-time”, an incidence density of 14.49 occurrences of DVWR was obtained for every 100 extubations, *per* day.

Most of the patients in the sample (52.7%) were female, with a mean age of 60.77 years old (SD ± 18.9), and variability from 18 to 96 years old, older adults being the most frequent age group (61.3%). Most of the patients were hospitalized for clinical reasons (73.1%), with sepsis being the most identified medical diagnosis in the sample. As for clinical severity, the mean SAPS 3 was 58.87 on admission to the ICU, which corresponds to a chance of death of approximately 45%, an estimate adjusted for Latin America^(^ 18 ^)^.

The main factor that motivated the use of MV was respiratory failure (52.7%), followed by sensory lowering (21.5%) and by surgery (14%). The mean MV time from the intubation date to the start of weaning was 4.45 days (SD ± 2.43). Regarding the time of MV until removal of the artificial airway (extubation), a mean of 7.09 days was observed (SD ± 4.29). Thus, the mean weaning time consisted of 2.64 days, i.e., 37.23% of the total mechanical ventilatory support time was allocated to the weaning process.

As for the ventilatory parameters programmed before extubation, FiO_2_ was adopted with a mean of 40%, as well as a PEEP value of 5 cmH_2_O and a support pressure of 10 cmH_2_O. The observed VT showed a mean of 450 mL/Kg and the median Vm was 8.61 mL/Kg. The median oxygenation index (PaO_2_/FiO_2_) was 300.

The mean time to extubation failure was 7.75 (SD ± 11.85) hours. Among the clinical situations considered motivating for extubation failure, acute respiratory failure was the most frequent cause among the patients (70.7%). Noninvasive ventilation was used for 33.3% of the individuals after extubation.

As for the clinical outcomes observed in the sample, 26.9% of the individuals underwent tracheostomy. VAP was diagnosed in 12.9% of the patients and death occurred in 32.3% of the cases.

There was a statistical association between the mean age and the occurrence of DVWR, with each year increasing in age, there is a 1.03-fold increase in the chance of developing the outcome (Table 1). For the gender and age group variables no significant association was found (p<0.05).

**Table 1:** Numerical variables related to extubated patients (n=93) statistically associated with the diagnosis of *Dysfunctional Ventilatory Weaning Response*(DVWR). Belo Horizonte, MG, Brazil, 2015-2016

Variables Mean	Extubation Success		Extubation Failure (DVWR)		p-value	OR (95% CI)
	Mean	SD	Mean	SD		
Age	56.85	18.48	65.76	18.53	0.027	1.03 (1.00-1.05)
SAPS 3*	54.52	13.13	64.39	17.06	0.004	1.04 (1.01-1.08)
Fluid balance Measure/100	1.64	13.04	13.04	13.14	0.000	1.08 (1.03-1.12)
MMSS Edema^†^	1.23	1.02	2.34	1.56	0.000	1.89 (1.34-2.66)
MMII Edema^‡^	1.02	0.94	2.39	1.56	0.000	2.21 (1.53-3.19)
Number of antibiotics	1.02	1.00	2.20	1.17	0.000	2.56
HR^§^	85.73	18.07	96.42	16.40	0.007	1.04 (1.01-1.06)

* SAPS 3 =*Simplified Acute Physiology Score*;

† MMSS = Upper limbs;

‡ MMII = Lower limbs;

§ HR = Heart rate

There were also no statistically significant differences between the extubation failure and success groups according to the type of hospitalization, main medical diagnoses (heart disease, sepsis, and gastrointestinal disease) and most frequent previous comorbidities, Chronic Obstructive Pulmonary Disease (COPD) and Systemic Arterial Hypertension (SAH), with a p-value greater than 0.05 in all cases. The same occurred for the weaning time, corresponding to the date of replacement of the controlled ventilation mode for an assisted mode until the day of extubation.

Clinical severity at admission was significantly associated with failure to wean, with patients with higher scores in the SAPS 3 values having a higher chance of occurrence of DVWR (Table 1).

The increase in heart rate was significantly associated with failure in extubation, increasing the chance of DVWR to 1.04 times for each unit increased in value (Table 1). Other vital signs such as respiratory rate, mean arterial pressure and peripheral oxygen saturation measured with an oximeter did not present statistically significant differences between the groups.

When analyzing the variables related to fluid balance, it was found that the more positive the fluid balance and edema of upper and lower limbs, the greater the chance of occurrence of DVWR. For every 100 units added to the fluid balance, there is an increase of 1.08 in the chance of extubation failure (Table 1). Additionally, with each point added to the locker value during the edema evaluation, there is an approximately 2-fold increase in the chance of the outcome occurring (Table 1). In addition, the presence of anasarca, occurrence of oliguria and hemodialysis increased the chance for the development of DVWR (Table 2).

**Table 2:** Categorical variables related to extubated patients (n=93) statistically associated with the diagnosis of Dysfunctional Ventilatory Weaning Response (DVWR) (n=93). Belo Horizonte, MG, Brazil, 2015-2016

Variables		Extubation Success		Extubation Failure (DVWR)		p-value	OR (95% CI)
		N	%	N	%		
Anasarca	No	50	62.0%	31	38.0%	0.001	-
	Yes	1	8.3%	11	91.7%		
Oliguria	No	47	75.8%	15	24.2%	00.000	1.00
	Yes	5	16.1%	26	83.9%		16.29 (5.32-49.93)
Hemodialysis	No	44	67.7%	21	32.3%	00.001	1.00
	Yes	8	28.6%	20	71.4%		5.24 (1.98-13.83)
Hyperthermia	No	47	66.2%	24	33.8%	00.001	1.00
	Yes	5	22.7%	17	77.3%		6.66 (2.19-20.24)
Hypothermia	No	50	61.7%	31	38.3%	0.004	-
	Yes	2	16.7%	10	83.3%		
Use of vasopressors	No	51	70.8%	21	29.2%	0.000	-
	Yes	1	4.8%	20	95.2%		
Muscular retraction	No	52	59.8%	35	40.2%		-
	Yes	0	0.0%	6	100 %		
Secretion amount	Mild	39	88.6%	5	11.4%	0.000	-
	Moderate	11	73.3%	4	26.7%		
	Abundant	2	5.9%	32	94.1%		
Anxiety score	Mild	37	97.4%	1	2.6%	0.000	-
	Moderate	13	56.5%	10	43.5%		
	Severe	2	6.3%	30	93.8%		
Use of NIV* after extubation	No	42	67.7%	20	32.3%	00.002	1.00
	Yes	10	32.3%	21	67.7%		4.41 (1.75-11.09)

* NIV = Non-Invasive Ventilation

It was observed that the greater the number of antibiotics administered, the greater the chance of developing DVWR, with each added antibiotic increasing the chance of DVWR by 2.6 times (Table 1). There was also an association between the occurrence of thermal changes, such as hyperthermia and hypothermia, and the occurrence of this ND (Table 2).

The administration of sedatives showed no statistical difference in relation to the outcome of extubation. In turn, the use of vasopressor, secretion amount in the orotracheal tube, presence of muscle retraction, and anxiety variables were statistically significant to the occurrence of DVWR (p<0.05) (Table 2). However, for these variables there were problems in estimating the OR, which was associated with the fact that they did not have one of the categories or had 2 or fewer patients in a certain category.

The oxygenation index and the performance of the SBT also did not show any association with the occurrence of DVWR.

Patients submitted to NIV after extubation, in turn, presented 4 times more extubation failure than those in which this ventilatory support was not implemented (Table 2).

There was no association between the occurrence of DVWR and the laboratory tests analyzed, such as gasometric parameters: pH, PaO_2_, pCO_2_, HCO_3_, BE, and SatO_2_, in addition to markers such as lactate, hemoglobin, hematocrit, creatinine, leukocytes and ions (sodium, magnesium, potassium, calcium and chlorine), all of which showed p values>0.05.

It is noteworthy that, despite having been identified as factors related to the occurrence of DVWR in the previous RIL, the following variables: Anionic Interval (GAP anion), type B Natriuretic Peptide (BNP), N-Terminal Fragment of type B Natriuretic Peptide (NT-proBNP), Albumin, Isovolumetric Relaxation Time, Central Volume of Carbon Dioxide (VCO_2_) and Central Oxygen Volume (VO_2_) were not analyzed, as the number of patients who performed these exams was small in the sample and because they are not routine tests performed in the services studied.

Among the clinical indicators present in patients with the ND of DVWR, tachypnea was observed more frequently after extubations (20.8%), followed by a drop in oxygen saturation and tachycardia (Table 3). It is worth mentioning that the patient could present more than one clinical indicator for determining DVWR.

**Table 3:** Occurrence of clinical indicators for determining *Dysfunctional Ventilatory Weaning Response*related to extubated patients (n=93). Belo Horizonte, MG, Brazil, 2015-2016

Variables	N	%
Tachypnea	36	20.8%
Drop in saturation	29	16.8%
Tachycardia	16	9.2%
Use of accessory muscles	13	7.5%
Restlessness	11	6.4%
Decrease in the level of consciousness	9	5.2%
Nostril throbbing	8	4.6%
Agitation	7	4.0%
Audible airway secretions	7	4.0%
Shallow breathing	6	3.5%
Laryngeal stridor	6	3.5%
Hypertension	5	2.9%
Wide eyes	4	2.3%
Impaired ability to cooperate	3	1.7%
Aprehension	2	1.2%
Gasping breaths	2	1.2%
Hyperfocused on activities	2	1.2%
Diaphoresis	1	0.6%
Fatigue	1	0.6%
Fear of machine malfunction	1	0.6%
Adventitious breath sounds	1	0.6%
Paradoxical abdominal breathing	1	0.6%
Feeling warm	1	0.6%
Perceived need for increase in oxygen	1	0.6%

Regarding the temporality of occurrence of the clinical DVWR indicators, tachypnea was more frequent (46.3%) in the first 30 minutes of monitoring (T1), followed by a drop in oxygen saturation at T2 (60 minutes) and by tachypnea at T3 (90 minutes). In the final three periods, nostril throbbing, use of accessory muscles and changes in the level of consciousness were the most frequent clinical manifestations at 120, 150 and 180 minutes, respectively. Table 4 shows the five most frequent clinical indicators for determining DVWR in each observation period.

**Table 4:** Temporal occurrence of the clinical indicators for determining *Dysfunctional Ventilatory Weaning Response*related to extubated patients (n=93). Belo Horizonte, MG, Brazil, 2015-2016

	Variables	N	%
**T1** **(n = 41)**	Tachypnea	19	46.3%
	Restlessness	5	12.2%
	Laryngeal stridor	4	9.8%
	Drop in saturation	3	7.3%
	Aprehension	2	4.9%
**T2** **(n = 35)**	Drop in saturation	8	22.9%
	Restlessness	4	11.4%
	Tachycardia	4	11.4%
	Tachypnea	4	11.4%
	Shallow breathing	3	8.6%
**T3** **(n = 26)**	Tachypnea	8	30.8%
	Tachycardia	5	19.2%
	Use of accessory muscles	3	11.5%
	Agitation	2	7.7%
	Drop in saturation	2	7.7%
**T4** **(n = 26)**	Nostril throbbing	5	19.2%
	Drop in saturation	4	15.4%
	Tachypnea	4	15.4%
	Audible airway secretions	3	11.5%
	Wide eyes	2	7.7%
**T5** **(n = 23)**	Use of accessory muscles	6	26.1%
	Drop in saturation	4	17.4%
	Tachycardia	3	13.0%
	Hypertension	3	13.0%
	Agitation	1	4.3%
**T6** **(n = 22)**	Decrease in the level of consciousness	7	31.8%
	Drop in saturation	5	22.7%
	Nostril throbbing	2	9.1%
	Laryngeal stridor	2	9.1%
	Hyperfocused on activities	2	9.1%

## Discussion

The process of interrupting mechanical ventilatory support is considered complex and liable to failure, making the removal of the patient from MV more difficult than maintaining it, which makes it difficult to determine the acceptable failure rate^(^ 19 ^)^. In this perspective, some authors describe weaning as a shady area of intensive care and that, even in specialized centers, can be considered a mixture of art and science^(^ 19 ^-^ 20 ^)^.

According to the Brazilian Intensive Care Association, despite protocols implemented in various services, extubation failure has occurred in about 24% of the cases in Brazil^(^ 21 ^)^. In the present study, the overall incidence of failure in ventilatory weaning, configuring the occurrence of DVWR, was 44.09%. It is worth noting that the higher incidence of the diagnosis identified can be attributed to the high clinical severity observed in the patients who composed the sample. This fact may be related to the low occurrence of admissions and elective intubations, since one of the hospitals included in the study is a reference center for trauma, urgency and emergency.

Previous studies revealed that the prevalence of patients who failed in the process of ventilatory weaning, even after extensive evaluation of the extubation potential, varied between 5% and 30%^(^ 19 ^,^ 21 ^-^ 26 ^)^. It should be noted that these results reflect a different reality from the Brazilian one, since they are mostly data from international studies.

It is estimated that, in order to make a more accurate diagnosis of dysfunctional responses to the process, the nurse will need evidence to support characteristic findings for the condition (signs and symptoms) and must be able to identify patients at risk of developing this ND to establish, together to the multi-professional team, preventive intervention actions^(^ 27 ^)^.

In this study, from a competing cohort, clinical markers were identified to determine the occurrence of failure in ventilatory weaning and its related factors, in order to clinically validate the ND of DVWR.

The characteristics of the studied sample have similarities and divergences when compared to those found in the literature. Regarding gender, there was a slight predominance of women (52.7%). However, no statistical difference (p<0.05) was found related to the variable, which confirms the findings of other authors^(^ 22 ^,^ 25 ^)^.

As for the age group, there was a higher frequency of elderly people in the sample (61.3%), with a mean age of 60.77 years old (SD ± 18.9). Nevertheless, there was no statistical difference between the age group and the occurrence of DVWR (p<0.05). However, a statistical association was observed between the mean age and the occurrence of DVWR (*p*=0.02), which corroborates the findings reported by previous investigations^(^ 25 ^,^ 28 ^-^ 29 ^)^. The association can be partially explained by the morphological and functional changes that occur in the respiratory system with aging, in addition to the greater number of comorbidities in this population.

Upon admission to the ICU, sepsis was the most identified medical diagnosis (25.8%), and SAH and COPD were the most frequent comorbidities in the sample. Although, in this study no statistically significant differences were identified between the extubation failure and success groups according to the medical diagnosis at admission and to the comorbidities. Some authors^(^ 25 ^,^ 29 ^-^ 31 ^)^ observed that sepsis, heart disease, obstructive pulmonary diseases and previous diseases such as cancer, hypertension and stroke were factors significantly associated with the occurrence of extubation failure, which may have not been identified in this research due to the small sample included or the clinical profile of the individuals studied.

Still on the clinical profile, it is worth noting that in this investigation it was decided to exclude patients with neurological disorders with the potential to affect the drive or ventilatory mechanics. The literature demonstrates that predicting extubation failure in this group of individuals is still a controversial topic that may be related to specific factors such as the inability to protect the airways, bulbar paralysis, respiratory failure caused by depression of the respiratory center, peripheral neuropathy or due to motor and cognitive sequelae that remain after the initial neurological condition and not just the pulmonary pathology^(^ 15 ^)^. Thus, many patients need to return to the orotracheal tube even after all traditional weaning parameters have been successfully met.

As for clinical severity, the SAPS 3 prognostic system was adopted, composed of twenty variables with different weights, divided into three different parts: demographic, physiological, and reasons for ICU admission. The variables give a score of 16 to 217 points that are proportional to the worst prognosis^(^ 32 ^)^. The patients followed up in this study had a mean SAPS 3 of 58.87 on admission to the ICU, which demonstrates the clinical severity and complexity of the nursing care required, since this value represents a chance of evolving to death of approximately 45%, an adjusted estimate for Latin America^(^ 18 ^)^. It was observed that patients with higher scores in the SAPS 3 values had a higher risk of occurrence of DVWR (p<0.05).

The use of SAPS 3 is able to show scores that infer prognosis beyond 24 hours of admission and reflect the complexity of the care demanded considering the patient’s clinical status^(^ 32 ^)^. Other authors^(^ 20 ^,^ 25 ^,^ 33 ^)^ also found an association between SAPS 3 and the occurrence of weaning failure, concluding that the SAPS 3 system has a good discriminatory power during the ventilatory weaning process.

It is noteworthy that aspects related to fluid balance proved to be decisive in the outcome of the weaning of the patients studied according to the results of this investigation. It was observed that the more positive the fluid balance and the greater the edema of upper and lower limbs, the greater the chance of occurrence of DVWR. The occurrence of oliguria and hemodialysis also increased the chance for the development of DVWR.

The association between positive fluid balance value and the consequent occurrence of oliguria with prolonged duration of MV weaning and extubation failure has been identified by other authors^(^ 34 ^)^. Besides, it was also demonstrated that the duration of MV and the time spent on weaning were significantly longer in patients with acute renal failure.

The exact role of decreased renal function on respiratory outcomes in critically ill patients is not yet fully elucidated, but it is suggested that this relationship can be partially explained by the interactions of fluids in respiratory muscle performance and lung volumes, which seems to be correlated with situations of systemic inflammation such as sepsis^(^ 35 ^)^. Thus, it is recommended that other cardiovascular factors be evaluated to analyze the value of hemodynamic monitoring and the role of diuretic therapy in preventing reintubation^(^ 29 ^)^.

Congestive heart failure has also been suggested as an important reason for the failure of weaning in patients with positive fluid balance, generally associated with increased pulmonary artery occlusion pressure^(^ 29 ^,^ 34 ^)^. This data may explain the fact that the heart rate variable has shown a statistically significant association in conjunction with oliguria and limb edema.

In this context, the nurse plays a fundamental role in the fluid control of critically ill patients, evaluating congestive signs such as the presence of edema, anasarca, and pulmonary crackles during physical examination and identifying signs such as decreased urine volume and increased renal slag.

Infection also stands out as an important factor related to delayed weaning from MV and to a worse prognosis for patients on MV^(^ 36 ^-^ 37 ^)^. It is considered that the associations observed between thermal changes and the number of antibiotics with the occurrence of DVWR may also be related to the context of the infection, since hyperthermia is considered a highly prevalent sign in the evolution of infectious conditions and the antibiotics are used in treatment.

It was found that patients undergoing NIV after extubation had 4 times more extubation failures. The implementation of this ventilatory support was also significantly associated in another investigation that evaluated 508 attempts at extubation and observed a 3.2 fold increase in the chance of failure when NIV was performed after the removal of the artificial airway^(^ 25 ^)^. The literature shows that the group that needs NIV after extubation has a significantly higher proportion of patients with chronic respiratory disease, which is related to a higher incidence of extubation failure in adjusted analyses^(^ 38 ^)^.

When analyzing the related factors described in the DVWR taxonomy, it is observed that, among the physiological factors described by NANDA-I^(^ 14 ^)^, *ineffective airway clearance*, assessed considering the amount of secretion in the airways, in fact showed a statistically significant association (p <0.05) with the outcome.

Of the psychological factors^(^ 14 ^)^, only anxiety was evaluated in this study, as it was the only aspect in which a quantitative analysis was possible through the application of a scale, enabling to conduct statistical tests to verify associations with the studied outcome. The STAI scale proposed by Spielberger in 1966 is a self-referred Likert-type scale containing 20 statements that should be graded in a range from 1 (absolutely not) to 4 (very much)^(^ 39 ^)^. However, this instrument was developed to be applied to patients with an adequate level of consciousness, orientation in time, space and the person himself, as well as the ability to communicate with the evaluator, which is generally not possible in critical patients undergoing mechanical ventilatory support.

In view of this limitation, an adaptation of the scale (STAI) was developed for application in patients undergoing MV^(^ 39 ^)^. Containing only six of the items on the original scale, the reduced version has the same psychometric properties as the STAI inventory, but with better applicability conditions, considering that critically ill patients have difficulties in answering extensive questionnaires^(^ 39 ^)^. In this study, the anxiety score measured by applying the STAI scale showed to be significantly associated (p<0.05) with DVWR.

Considering the situational factors described in NANDA-I^(^ 14 ^)^: environmental barrier, uncontrolled episodic energy demands, inappropriate pace in decreasing ventilatory weaning, and insufficient social support, it is highlighted that these were not evaluated in this investigation due to the difficulty of measuring these variables for the type of idealized clinical validation study.

Among the associated conditions mentioned by NANDA-I^(^ 14 ^)^, the *history of ventilator dependence for more than 4 days* was analyzed considering the MV time variable; however, there was no statistically significant association between the time the patient spent on MV before the start of weaning or extubation and the outcome of ventilatory weaning. In this study, the weaning time corresponding to the replacement date of the controlled ventilation mode for an assisted modality until the day of extubation consisted of 37.32% of the total MV time. This result was similar to other studies that estimate that the process of MV removal takes up about half of the total time of ventilatory support^(^ 40 ^-^ 42 ^)^. However, both the weaning time and the total MV time, concerning the date of intubation until the day of extubation, did not show statistically significant differences between the groups.

The other associated condition described in the taxonomy, *history of unsuccessful weaning attempts*, was analyzed based on the performance of the SBT variable, since previous extubation failure was adopted as an exclusion criterion in this investigation. However, the SBT was also not statistically associated with the occurrence of DVWR.

In this study, among the clinical indicators present in the ND of DVWR, proposed by NANDA-I^(^ 14 ^)^ and those identified in the studies selected in the ILR, tachypnea was observed more frequently after extubations, followed by a drop in oxygen saturation and tachycardia, which corroborates the previous findings which report that post-extubation respiratory failure, expressed by visible signs of increased respiratory effort, is a common event and is associated with increased morbidity and mortality in the ICU^(^ 22 ^,^ 43 ^)^.

The defining characteristics of this ND were the subject of a study conducted to verify the temporality occurrence of these clinical indicators. It was found that 18% of the defining characteristics proposed by the taxonomy occurred in the first 30 minutes of observation, and it is possible to classify these as short duration events, indicating the need to return to the ventilatory prosthesis, with sufficient severity to motivate the nursing team to interrupt the MV withdrawal process^(^ 17 ^)^. The existence of temporal patterns of the defining characteristics of the ND of DVWR was also confirmed through the findings of this study, identifying progression of the severity of clinical manifestations in relation to the time of observation.

It was found that tachypnea and drop in saturation occurred more frequently in the initial times and, in the final three periods, signs and symptoms considered more serious occurred, such as nostril throbbing, use of accessory muscles, and changes in the level of consciousness, showing the progression of the installed respiratory failure. Such findings highlight the need for nurses to direct their attention from the first minutes of interrupting ventilation to identify the first signs of DVWR.

With these results, it can be inferred that the assessment made by the nurse during the phase of MV interruption in the process of ventilatory weaning should indicate, starting as soon as possible with the removal of the prosthesis, since the first signs and symptoms of failure can be identified almost immediately.

During the ventilatory weaning process, patients are assessed for their ability to breathe spontaneously and remove the artificial airway. Thus, the assistance provided to mechanically ventilated patients must be multi-professional, individualized and based on scientific evidence, in order to minimize the negative repercussions related to failure in the ventilatory weaning process, in addition to the unfavorable clinical outcomes intrinsic to prolonged MV.

In this sense, the role of nurses is of fundamental importance for the early and accurate performance of weaning from MV, as well as in the implementation of various care for mechanically ventilated patients, which precede the weaning process^(^ 13 ^)^.

Therefore, it is imperative that nurses working in the ICU are qualified to provide patient care in mechanical ventilatory support, including monitoring of ventilatory parameters and acknowledging alarms; mobilization, removal, and characterization of secretions; heating and humidifying the inhaled gases; positioning actions that consider the optimization of gas exchanges; performing specific oral hygiene in patients with orotracheal tube and tracheostomy; continuous assessment of the state of consciousness or level of sedation, as well as participating with the multi-professional team in the process of ventilatory weaning and removal of the artificial airway and the nurse is also an important trigger for the discussion to start the MV interruption process.

Therefore, in view of the findings of this study, it is observed that there are both defining characteristics and factors related to the problem available in the ND proposed by NANDA-I^(^ 14 ^)^ and that could be clinically validated in this investigation, as there are clinical variables, identified in the literature and proven in this sample, which are not yet described in the taxonomy, which points to the need to review this ND.

It is known that any and all research work has limitations that may be linked to some aspects, be it the method, the researcher, the individuals, the costs, and the very process of building specific knowledge, among others. Thus, this study is no exception. Although the accuracy of the ND of DVWR contributes with originality and relevance, the study has limitations such as: the small number of patients presented in the sample, which may have limited the verification of associations of the ND to other independent variables analyzed; the bedside follow-up method, which can be long and exhaustive, especially when evaluating subjective variables, such as anxiety and some clinical indicators for determining the occurrence of the diagnosis, allowing for omission errors in data collection.

It is a fact that other studies should be carried out to complement, confront, and/or to corroborate the results discussed in this investigation. Although the study was carried out in four ICU from two hospitals with different clinical profiles, as a strategy in an attempt to increase the potential of extrapolating this research, the need to expand the investigation in order to legitimize external validity, is promising to validate the diagnosis in a more heterogeneous population. Thus, it is suggested to carry out studies that evaluate a greater number of extubation events, in addition to the extension to samples other than the ones observed in this research and for services in which other models of weaning protocols are used.

It is also recommended to investigate other related factors described in NANDA-I^(^ 14 ^)^ for the diagnosis and which could not be validated in this study, such as the physiological factors: alterations in sleep patterns, pain and inadequate nutrition; the psychological factors: low self-esteem, insufficient trust in health professional, insufficient knowledge about the weaning process, hopelessness, feeling of helplessness, uncertainty about ability to wean, fear, decreased motivation and the situational factors: environmental barrier, uncontrolled episodic energy demands, history of unsuccessful weaning attempts, inappropriate pace in reducing ventilatory weaning and insufficient social support.

Finally, it is suggested to propose a ND of *Risk for the Occurrence of DVWR*, recognizing potential factors for its development, in order to establish strategies to prevent the problem among critically ill patients undergoing ventilatory weaning.

It is believed that this study may contribute to the improvement in the management of ventilatory weaning of patients admitted to an adult ICU, in addition to boosting the development of future studies on a theme that is still little explored by the nursing team, collaborating to improve the accuracy of the clinical judgments about real or potential problems of ventilatory weaning and implementation of nursing interventions to improve the quality of care provided to mechanically ventilated patients.

## Conclusion

The realization of this study made it possible to estimate the incidence of the ND of *Dysfunctional Ventilatory Weaning Response* (DVWR) among adult patients admitted to the ICU of teaching hospitals in Belo Horizonte - MG, in addition to knowing the clinical indicators for determining its occurrence and the factors related to its development in this sample.

Therefore, recommendations can be outlined for further studies and development of the ND. It is suggested that changes be made to the ND of DVWR proposed by NANDA-I, with the inclusion of other related factors such as: age; clinical severity on admission to the ICU (SAPS 3); disturbances of fluid balance (fluid balance value, occurrence of oliguria, hemodialysis, presence of edema in the upper and/or lower limbs); ongoing infectious conditions (quantity of antibiotics administered, occurrence of hyperthermia); hemodynamic changes (elevated heart rate, use of vasopressors) and, finally, use of NIV after extubation.
